# The genome sequence of the harlequin ladybird, *Harmonia axyridis *(Pallas, 1773)

**DOI:** 10.12688/wellcomeopenres.17349.3

**Published:** 2024-11-28

**Authors:** Douglas Boyes, Liam M. Crowley, University of Oxford and Wytham Woods Genome Acquisition Lab, Darwin Tree of Life Barcoding collective, Wellcome Sanger Institute Tree of Life programme, Wellcome Sanger Institute Scientific Operations: DNA Pipelines collective, Tree of Life Core Informatics collective, Darwin Tree of Life Consortium

**Affiliations:** 1 UK Centre for Ecology & Hydrology, Wallingford, UK; 2 Department of Zoology, University of Oxford, Oxford, UK

**Keywords:** Harmonia axyridis, harlequin ladybird, genome sequence, chromosomal

## Abstract

We present a genome assembly from an individual female *Harmonia axyridis* (the harlequin ladybird; Arthropoda; Insecta; Coleoptera; Coccinellidae). The genome sequence is 426 megabases in span. The majority (99.98%) of the assembly is scaffolded into 8 chromosomal pseudomolecules, with the X sex chromosome assembled.

## Species taxonomy

Eukaryota; Metazoa; Ecdysozoa; Arthropoda; Hexapoda; Insecta; Pterygota; Neoptera; Endopterygota; Coleoptera; Polyphaga; Cucujiformia; Coccinellidae; Coccinellinae; Coccinellini; Harmonia; *Harmonia axyridis* (Pallas, 1773) (NCBI:txid115357).

## Background

The harlequin ladybird, *Harmonia axyridis*, is large (5–8 mm), voracious ladybird species widely considered to be one of the world’s most invasive insects. Its native range is central and eastern Asia, but it was introduced to North America and Europe as a biocontrol agent. It has spread rapidly and is now established across North, Central and South America, Europe and Africa. Examination of microsatellites has demonstrated that an invasive population in eastern North America acted as the source of those which invaded Europe, South America and South Africa ( Lombaert *et al*., 2010). The Harlequin ladybird was first recorded in the UK in 2003 in south-eastern England. Since its arrival it spread rapidly and is now widespread across the UK, and has been recorded on Ireland, Orkney, Shetland, the Channel Islands, the Isles of Scilly and the Isle of Man. It is a highly polymorphic species with several recognised forms. The colour of the elytra ranges from yellow, orange, red or black, with 0–21 black spots, 4 or 2 red/orange spots. The legs are always brown and the underside is dark with a reddish/brown border. The harlequin ladybird is a generalist, feeding on aphids as well as soft fruit, pollen, nectar and many other soft-bodied insects, including other ladybird larvae. It overwinters as an adult and is often found in buildings where aggregations of adults form. The haemolymph of this species contains high concentrations of isopropyl methoxy pyrazine ( Al Abassi *et al*., 1998) and harmonine ( Nagel *et al*., 2015) and it readily autohaemorrhages when agitated. The defensive secretions have a foul odour and can cause staining. Furthermore, it is also known to bite humans ( Ramsey & Losey, 2012), leading to this species’ consideration as a minor household pest. The spread of the harlequin ladybird is associated with dramatic declines in other, native ladybird species. This is believed to be driven by *Harmonia axyridis* outcompeting other aphidophagous species as well as intraguild predation ( Majerus *et al*., 2006).

## Genome sequence report

**Figure 1.**
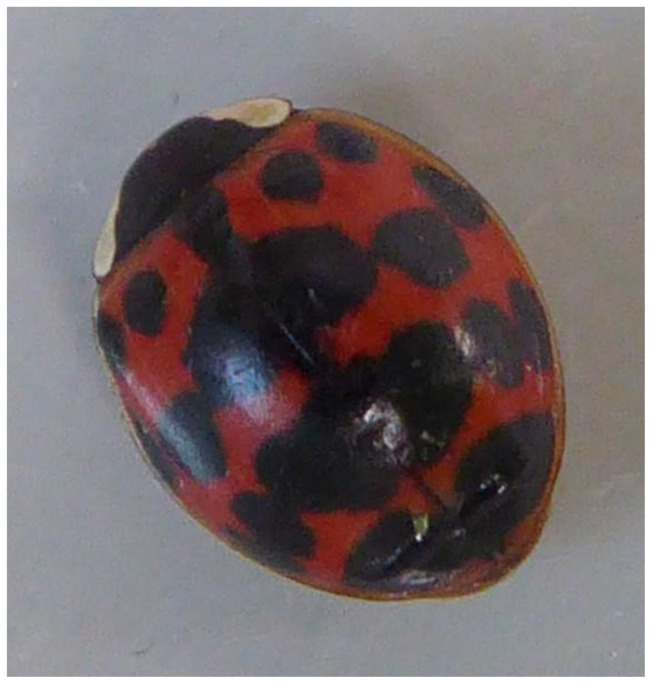
An image of the sequenced specimen, icHarAxyr1, captured immediately prior to processing and preservation.

The genome was sequenced from one female *H. axyridis* collected from Wytham Woods, Oxfordshire (biological vice-county: Berkshire), UK (latitude 51.772, longitude –1.338) ( Figure 1). A total of 53-fold coverage in Pacific Biosciences single-molecule long reads and 93-fold coverage in 10X Genomics read clouds were generated. Primary assembly contigs were scaffolded with chromosome conformation Hi-C data. Manual assembly curation corrected 158 missing/misjoins, reducing the assembly length by 1.32% and the scaffold number by 92.49%, and increasing the scaffold N50 by 56.15%.

**Figure 2.**
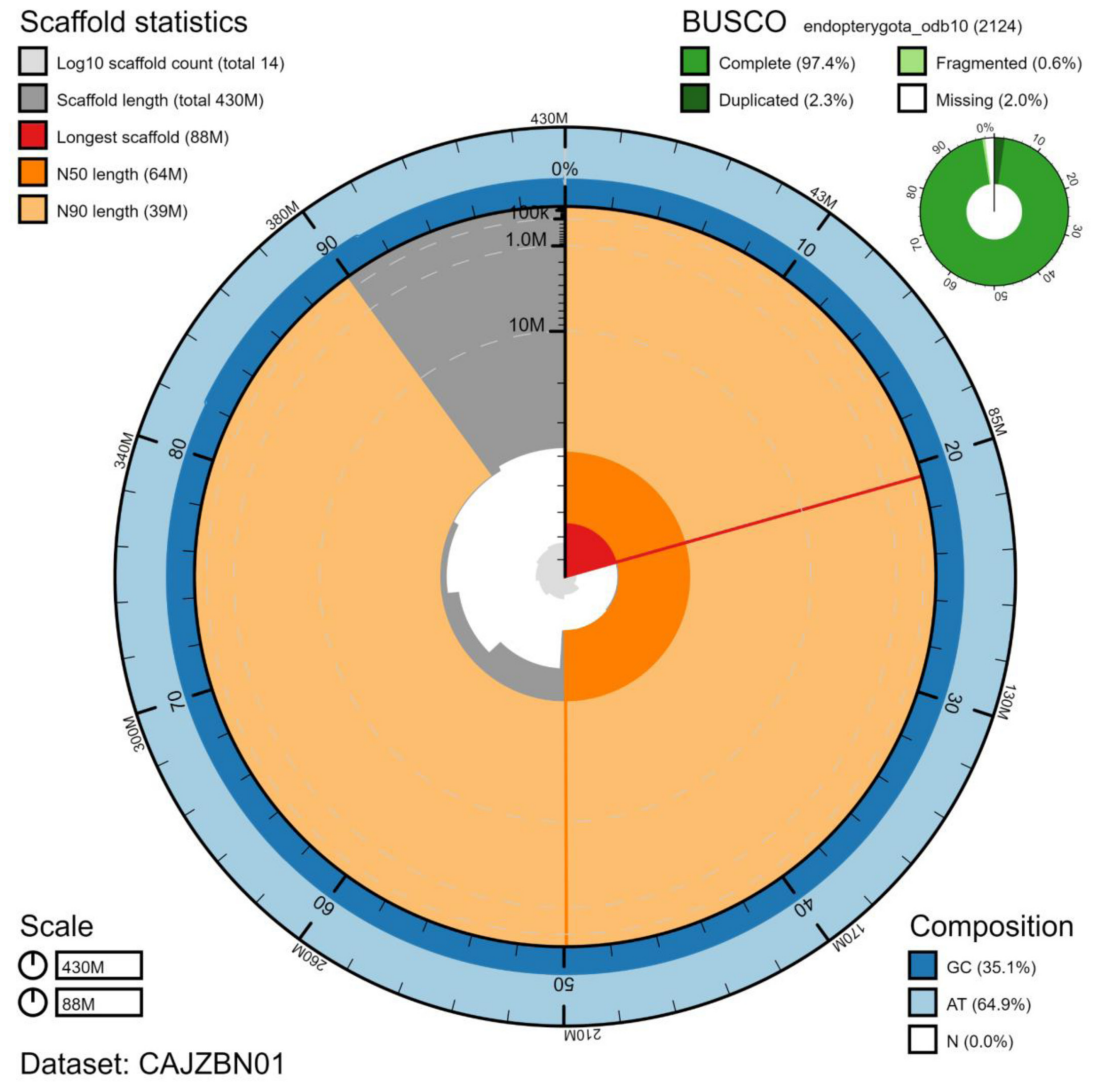
Genome assembly of *Harmonia axyridis*, icHarAxyr1.1: metrics. The BlobToolKit Snailplot shows N50 metrics and BUSCO gene completeness. The main plot is divided into 1,000 size-ordered bins around the circumference with each bin representing 0.1% of the 425,544,856 bp assembly. The distribution of scaffold lengths is shown in dark grey with the plot radius scaled to the longest scaffold present in the assembly (87,845,136 bp, shown in red). Orange and pale-orange arcs show the N50 and N90 scaffold lengths (63,675,256 and 38,596,305 bp), respectively. The pale grey spiral shows the cumulative scaffold count on a log scale with white scale lines showing successive orders of magnitude. The blue and pale-blue area around the outside of the plot shows the distribution of GC, AT and N percentages in the same bins as the inner plot. A summary of complete, fragmented, duplicated and missing BUSCO genes in the endopterygota_odb10 set is shown in the top right. An interactive version of this figure is available at https://blobtoolkit.genomehubs.org/view/icHarAxyr1.1/dataset/CAJZBN01/snail.

The final assembly has a total length of 425.5 Mb in 13 sequence scaffolds with a scaffold N50 of 63.7 Mb ( Table 1). Most of the assembly sequence (99.98%) was assigned to 8 chromosomal-level scaffolds, representing 7 autosomes (numbered by sequence length), and the X sex chromosome ( Figure 2– Figure 5; Table 2). Some scaffolds remain unplaced due to repetitive content giving an ambiguous Hi-C signal. A large cluster of rDNA sequences was placed on the X chromosome using Hi-C data only. The assembly has a BUSCO v5.1.2 ( Manni *et al*., 2021) completeness of 97.4% (single 95.2%, duplicated 2.3%) using the endopterygota_odb10 reference set. While not fully phased, the assembly deposited is of one haplotype. Contigs corresponding to the second haplotype have also been deposited.

**Table 1.** Genome data for *Harmonia axyridis*, icHarAxyr1.1.

*Project accession data*	
Assembly identifier	icHarAxyr1.1
Species	*Harmonia axyridis*
Specimen	icHarAxyr1
NCBI taxonomy ID	NCBI:txid346838
BioProject	PRJEB45202
BioSample ID	SAMEA7520208
Isolate information	Female, whole organism
*Raw data accessions*	
PacificBiosciences SEQUEL II	ERR6565943
10X Genomics Illumina	ERR6054990-ERR6054993
Hi-C Illumina	ERR6054994
*Genome assembly*	
Assembly accession	GCA_914767665.1
Accession of alternate haplotype	GCA_914767675.1
Span (Mb)	426
Number of contigs	186
Contig N50 length (Mb)	22.9
Number of scaffolds	13
Scaffold N50 length (Mb)	63.7
Longest scaffold (Mb)	87.9
BUSCO * genome score	C:97.4%[S:95.2%,D:2.3%],F:0.6%,M:2.0%,n:2,124

*BUSCO scores based on the endopterygota_odb10 BUSCO set using v5.1.2. C= complete [S= single copy, D=duplicated], F=fragmented, M=missing, n=number of orthologues in comparison. A full set of BUSCO scores is available at https://blobtoolkit.genomehubs.org/view/icHarAxyr1.1/dataset/CAJZBN01/busco.

**Figure 3.**
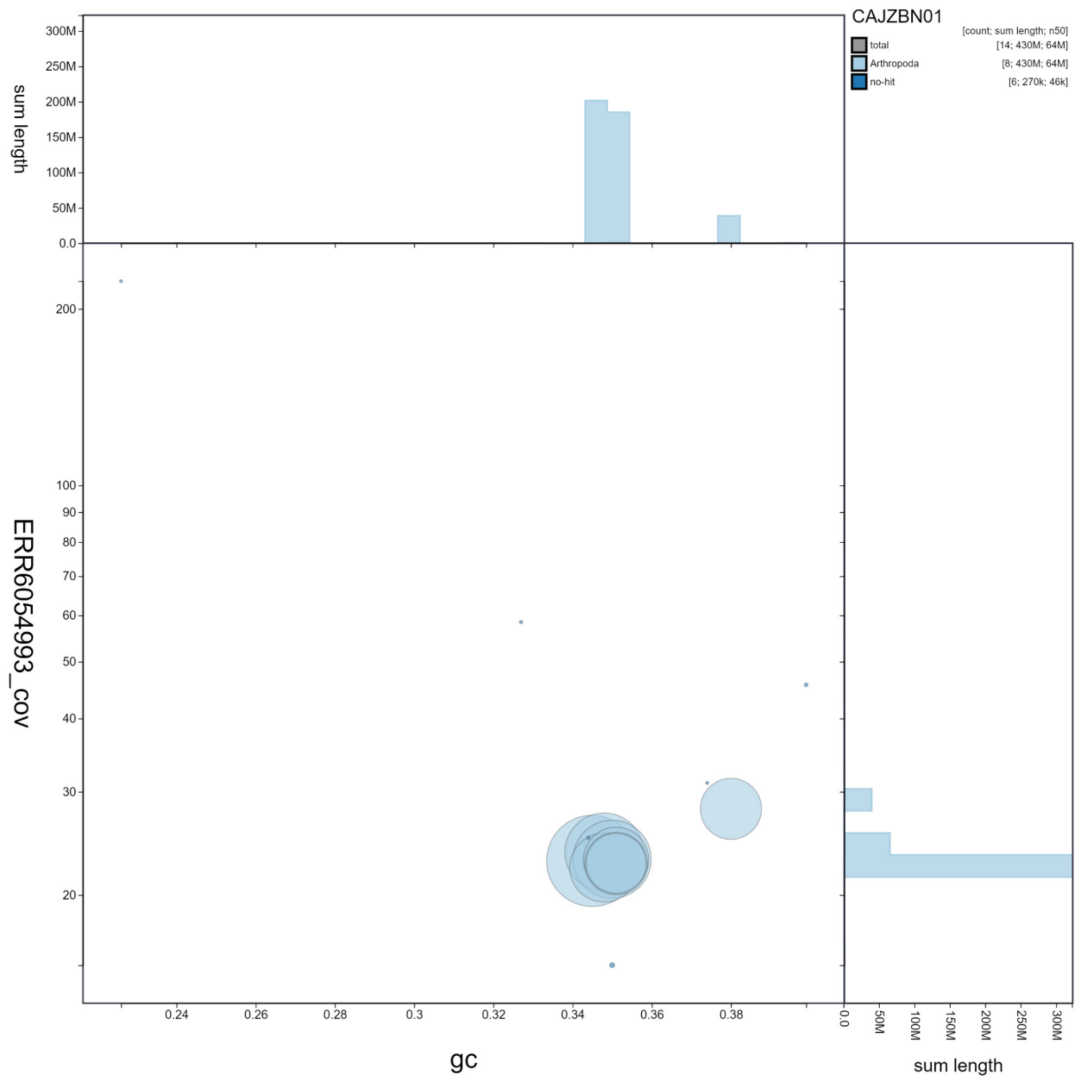
Genome assembly of *Harmonia axyridis*, icHarAxyr1.1: GC coverage. BlobToolKit GC-coverage plot. Scaffolds are coloured by phylum. Circles are sized in proportion to scaffold length Histograms show the distribution of scaffold length sum along each axis. An interactive version of this figure is available at https://blobtoolkit.genomehubs.org/view/icHarAxyr1.1/dataset/CAJZBN01/blob.

**Figure 4.**
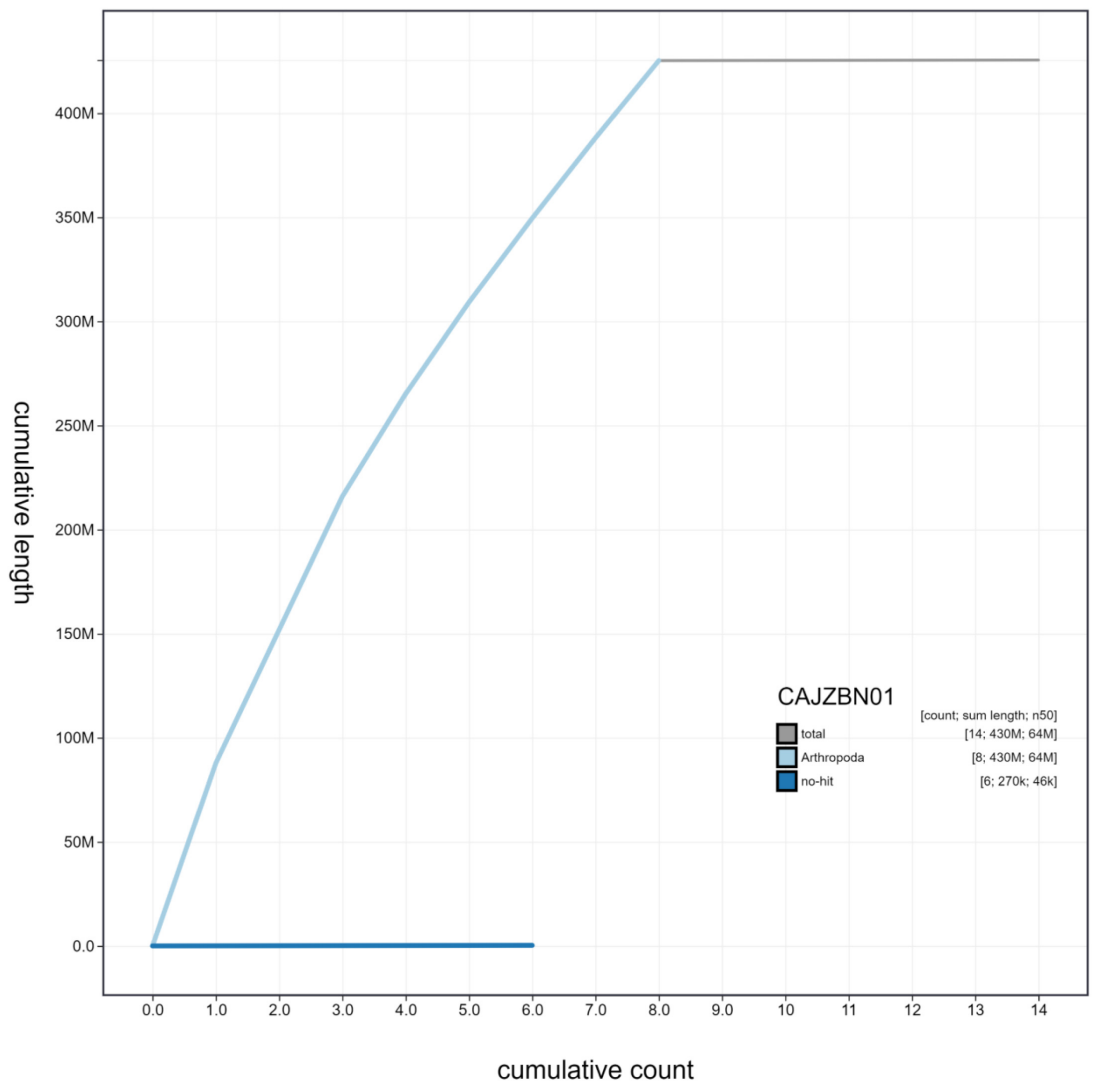
Genome assembly of *Harmonia axyridis*, icHarAxyr1.1: cumulative sequence. BlobToolKit cumulative sequence plot. The grey line shows cumulative length for all scaffolds. Coloured lines show cumulative lengths of scaffolds assigned to each phylum using the buscogenes taxrule. An interactive version of this figure is available at https://blobtoolkit.genomehubs.org/view/icHarAxyr1.1/dataset/CAJZBN01/cumulative.

**Figure 5.**
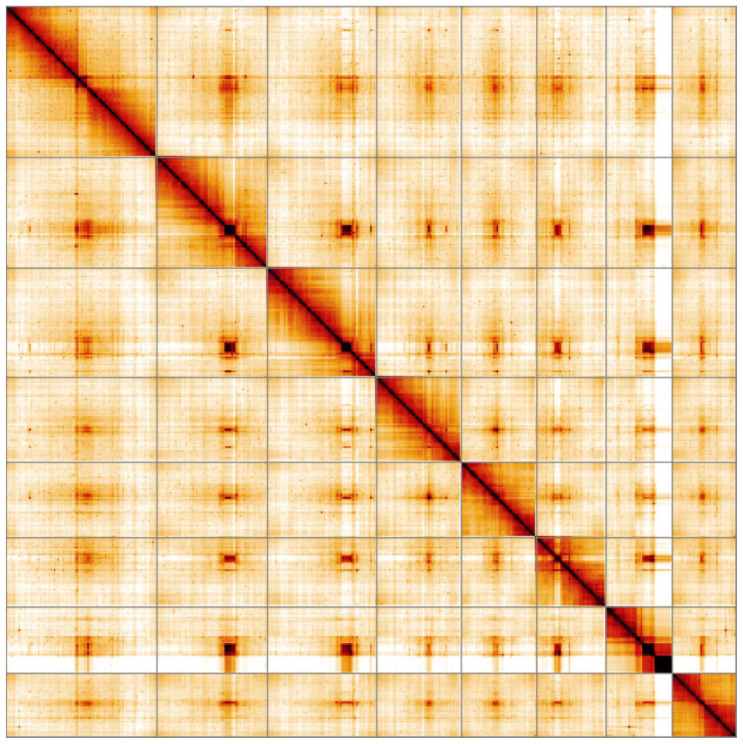
Genome assembly of *Harmonia axyridis*, icHarAxyr1.1: Hi-C contact map. Hi-C contact map of the icHarAxyr1.1 assembly, visualised in HiGlass.

**Table 2.** Chromosomal pseudomolecules in the genome assembly of *Harmonia axyridis*, icHarAxyr1.1.

INSDC accession	Chromosome	Size (Mb)	GC%
OU611927.1	1	87.85	34.5
OU611928.1	2	64.43	34.8
OU611929.1	3	63.68	35
OU611930.1	4	49.28	34.8
OU611931.1	5	43.98	35.1
OU611932.1	6	40.34	35.1
OU611934.1	7	37.13	35.1
OU611933.1	X	38.60	38
OU611935.1	MT	0.02	22.6
-	Unplaced	0.25	35.7

## Methods

### Sample acquisition, DNA extraction and sequencing

A single female *H. axyridis* was collected from Wytham Woods, Oxfordshire (biological vice-county: Berkshire), UK (latitude 51.772, longitude -1.338) by Douglas Boyes, UKCEH, using a pooter. The sample was identified by the same individual, and preserved on dry ice.

DNA was extracted from the whole organism of *H. axyridis* (icHarAxyr1) at the Wellcome Sanger Institute Scientific Operations core from the whole organism using the Qiagen MagAttract HMW DNA kit, according to the manufacturer’s instructions. Pacific Biosciences HiFi circular consensus and 10X Genomics read cloud DNA sequencing libraries were constructed according to the manufacturers’ instructions. Sequencing was performed by the Scientific Operations core on Pacific Biosciences SEQUEL II and Illumina HiSeq X instruments. Hi-C data were generated from the whole organism using the Arima v2 Hi-C kit and sequenced on a HiSeq X instrument.

All Wellcome Sanger Tree of Life wet lab protocols are available at DOI: 10.17504/protocols.io.8epv5xxy6g1b/v1.

### Genome assembly

Assembly was carried out with Hifiasm ( Cheng *et al*., 2021) using the --primary option; haplotypic duplication was identified and removed with purge_dups ( Guan *et al*., 2020). One round of polishing was performed by aligning 10X Genomics read data to the assembly with LongRanger align, calling variants with Freebayes ( Garrison & Marth, 2012). The assembly was then scaffolded with Hi-C data ( Rao *et al*., 2014) using SALSA2 ( Ghurye *et al*., 2019). The assembly was checked for contamination and corrected using the gEVAL system ( Chow *et al*., 2016) as described previously ( Howe *et al*., 2021). Manual curation ( Howe *et al*., 2021) was performed using gEVAL, HiGlass ( Kerpedjiev *et al*., 2018) and Pretext. The mitochondrial genome was assembled using MitoHiFi ( Uliano-Silva *et al*., 2021). The genome was analysed and BUSCO scores generated within the BlobToolKit environment ( Challis *et al*., 2020). The assembly pipelines are available at https://pipelines.tol.sanger.ac.uk/pipelines and https://github.com/sanger-tol/. Table 3 contains a list of all software tool versions used, where appropriate.

**Table 3.** Software tools used.

Software tool	Version	Source
Hifiasm	0.12	Cheng *et al*., 2021
purge_dups	1.2.3	Guan *et al*., 2020
SALSA2	2.2	Ghurye *et al*., 2019
longranger align	2.2.2	https://support.10xgenomics.com/genome- exome/software/pipelines/latest/advanced/ other-pipelines
freebayes	1.3.1-17-gaa2ace8	Garrison & Marth, 2012
MitoHiFi	1.0	Uliano-Silva *et al*., 2021
gEVAL	N/A	Chow *et al*., 2016
HiGlass	1.11.6	Kerpedjiev *et al*., 2018
PretextView	0.2.x	https://github.com/wtsi-hpag/PretextView
BlobToolKit	2.6.2	Challis *et al*., 2020

### Ethics/compliance issues

The materials that have contributed to this genome note have been supplied by a Darwin Tree of Life Partner. The submission of materials by a Darwin Tree of Life Partner is subject to the Darwin Tree of Life Project Sampling Code of Practice. By agreeing with and signing up to the Sampling Code of Practice, the Darwin Tree of Life Partner agrees they will meet the legal and ethical requirements and standards set out within this document in respect of all samples acquired for, and supplied to, the Darwin Tree of Life Project. Each transfer of samples is further undertaken according to a Research Collaboration Agreement or Material Transfer Agreement entered into by the Darwin Tree of Life Partner, Genome Research Limited (operating as the Wellcome Sanger Institute), and in some circumstances other Darwin Tree of Life collaborators.

## Data Availability

European Nucleotide Archive: Harmonia axyridis (harlequin). Accession number PRJEB45202; https://identifiers.org/ena.embl/PRJEB45202. The genome sequence is released openly for reuse. The *H. axyridis* genome sequencing initiative is part of the Darwin Tree of Life (DToL) project. All raw sequence data and the assembly have been deposited in INSDC databases. The genome will be annotated and presented through the Ensembl pipeline at the European Bioinformatics Institute. Raw data and assembly accession identifiers are reported in Table 1.

## Author information

Members of the University of Oxford and Wytham Woods Genome Acquisition Lab are listed here: https://doi.org/10.5281/zenodo.4789929.

Members of the Darwin Tree of Life Barcoding collective are listed here: https://doi.org/10.5281/zenodo.4893704.

Members of the Wellcome Sanger Institute Tree of Life programme collective are listed here: https://doi.org/10.5281/zenodo.5377053.

Members of Wellcome Sanger Institute Scientific Operations: DNA Pipelines collective are listed here: https://doi.org/10.5281/zenodo.4790456.

Members of the Tree of Life Core Informatics collective are listed here: https://doi.org/10.5281/zenodo.5013542.

Members of the Darwin Tree of Life Consortium are listed here: https://doi.org/10.5281/zenodo.4783559.
