# Supplementary material for: The genome sequence of the harlequin ladybird, Harmonia axyridis (Pallas, 1773)
Source: Wellcome Open Res. 2024 Nov 28;6:300. Originally published 2021 Nov 8. [Version 3] doi: 10.12688/wellcomeopenres.17349.3 (PMC11589414; doi:10.12688/wellcomeopenres.17349.3)
Supplement: Peer review history

# Reviewer report: Reviewer response for version 3

**DOI:** 10.21956/wellcomeopenres.25898.r113907

**Published:** 2024-12-16

**Authors:** Manee M Manee

**Affiliations:** 1 King Abdulaziz City for Science and Technology, Riyadh, Saudi Arabia

The paper presents a high-quality chromosome-level genome assembly of the harlequin ladybird ( *Harmonia axyridis*). The assembly spans 426 Mb, with 99.98% scaffolded into eight chromosomal pseudomolecules, achieving a completeness score of 97.4% using BUSCO. I am satisfied with the revised version of the manuscript after addressing the other reviewers' comments.

Are sufficient details of methods and materials provided to allow replication by others?

Yes

Is the rationale for creating the dataset(s) clearly described?

Yes

Are the datasets clearly presented in a useable and accessible format?

Yes

Are the protocols appropriate and is the work technically sound?

Yes

Reviewer Expertise:

bioinformatics, genomics, evolutionary biology

I confirm that I have read this submission and believe that I have an appropriate level of expertise to confirm that it is of an acceptable scientific standard.

# Reviewer report: Reviewer response for version 3

**DOI:** 10.21956/wellcomeopenres.25898.r113705

**Published:** 2024-12-02

**Authors:** Yu-Hao Huang

**Affiliations:** 1 Guangdong Key Laboratory of Animal Conservation and Resource Utilization, Guangdong Public Laboratory of Wild Animal Conservation and Utilization, Institute of Zoology, Guangdong Academy of Sciences, Guangzhou, China

The revised version has addressed my primary concern, and I have no further comments.

Are sufficient details of methods and materials provided to allow replication by others?

Yes

Is the rationale for creating the dataset(s) clearly described?

Yes

Are the datasets clearly presented in a useable and accessible format?

Yes

Are the protocols appropriate and is the work technically sound?

Yes

Reviewer Expertise:

comparative genomics, evolutionary biology, bioinformatics, phylogenetics, ladybird beetle

I confirm that I have read this submission and believe that I have an appropriate level of expertise to confirm that it is of an acceptable scientific standard.

# Reviewer report: Reviewer response for version 3

**DOI:** 10.21956/wellcomeopenres.25898.r113707

**Published:** 2024-12-02

**Authors:** Sudhakar Sivasubramaniam, Arun Arumugaperumal

**Affiliations:** 1 Manonmaniam Sundaranar University, Tirunelveli, Tamil Nadu, India; 2 Department of Biotechnology, Rajalakshmi Engineering College, Thandalam, Chennai, Tamilnadu, 602105, India

The authors have corrected the assembly parameters in text as per Table 1. The data note can be accepted.

Are sufficient details of methods and materials provided to allow replication by others?

Yes

Is the rationale for creating the dataset(s) clearly described?

Yes

Are the datasets clearly presented in a useable and accessible format?

Yes

Are the protocols appropriate and is the work technically sound?

Yes

Reviewer Expertise:

Molecular Biology, Transcriptomics, Genomics, Regeneration biology

We confirm that we have read this submission and believe that we have an appropriate level of expertise to confirm that it is of an acceptable scientific standard.

# Reviewer report: Reviewer response for version 2

**DOI:** 10.21956/wellcomeopenres.23551.r112729

**Published:** 2024-11-25

**Authors:** Yu-Hao Huang

**Affiliations:** 1 Guangdong Key Laboratory of Animal Conservation and Resource Utilization, Guangdong Public Laboratory of Wild Animal Conservation and Utilization, Institute of Zoology, Guangdong Academy of Sciences, Guangzhou, China

The data note presents a high-quality chromosome-level genome assembly of the ladybird beetle *Harmonia axyridis*, demonstrating high completeness and continuity that surpasses other published genomes of this species. This genome assembly will be instrumental for appropriate utilization of this potential biocontrol agent and the mitigation of its impact in introduced regions. The sequencing and bioinformatics methods are sound, and the data is accessible.

 However, before approving this version of the manuscript, I have a major concern. The descriptive text of the genome assembly (total length: 249 Mb, scaffold number: 39, scaffold N50: 37.2 Mb, 10 or 9? chromosomal-level scaffolds including 8 autosomes and the X sex chromosome) seems different with Tables 1 and 2 (total length: 426 Mb, scaffold number: 13, scaffold N50: 63.7 Mb, 7 autosomes and the X sex chromosome). Based on my experience of using this genome assembly, the information in the tables is closer to the actual one. Please verify and correct the descriptive text if necessary, or provide an explanation for the discrepancy (e.g., before and after curation).

 Additionally, there are at least three previously published genomes of *H. axyridis* (Ando, et al. 2018; Gautier, et al. 2018; Chen, et al. 2021). It is better to mention these genomes in the background to provide readers with a better understanding of the current genome assembly.

Are sufficient details of methods and materials provided to allow replication by others?

Yes

Is the rationale for creating the dataset(s) clearly described?

Yes

Are the datasets clearly presented in a useable and accessible format?

Yes

Are the protocols appropriate and is the work technically sound?

Yes

Reviewer Expertise:

comparative genomics, evolutionary biology, bioinformatics, phylogenetics, ladybird beetle

I confirm that I have read this submission and believe that I have an appropriate level of expertise to confirm that it is of an acceptable scientific standard, however I have significant reservations, as outlined above.

## References

[1] : Repeated inversions within a pannier intron drive diversification of intraspecific colour patterns of ladybird beetles. *Nat Commun* .2018;9(1) : 10.1038/s41467-018-06116-1 3843 10.1038/s41467-018-06116-1 30242156 PMC6155092

[2] : The Genomic Basis of Color Pattern Polymorphism in the Harlequin Ladybird. *Curr Biol* .2018;28(20) : 10.1016/j.cub.2018.08.023 3296-3302.e7 10.1016/j.cub.2018.08.023 30146156 PMC6203698

[3] : A chromosome-level assembly of the harlequin ladybird Harmonia axyridis as a genomic resource to study beetle and invasion biology. *Mol Ecol Resour* .2021;21(4) : 10.1111/1755-0998.13342 1318-1332 10.1111/1755-0998.13342 33529495

# Reviewer report: Reviewer response for version 2

**DOI:** 10.21956/wellcomeopenres.23551.r106346

**Published:** 2024-11-07

**Authors:** Sudhakar Sivasubramaniam, Arun Arumugaperumal

**Affiliations:** 1 Manonmaniam Sundaranar University, Tirunelveli, Tamil Nadu, India; 2 Department of Biotechnology, Rajalakshmi Engineering College, Thandalam, Chennai, Tamilnadu, 602105, India

The authors have sequenced the genome of the ladybird beetle, *Harmonia axyridis*. Already a genome sequence for the insect is available [1]. This assembly has a higher N50 value compared to the previously reported one. It is good to have an improved assembly. But the authors should cite the already published article and mention this. Then only readers can easily understand the difference between the assemblies. Ignoring the already published article may lead to confusion. 

 Also I have few suggestions,

 1. In Background section, the line "The final assembly has a total length of 249 Mb in 39 sequence scaffolds with a scaffold N50 of 37.2 Mb (Table 1)." mentions that scaffold N50 is 37.2 Mb and refers table 1. But in Table 1 scaffold N50 is mentioned as 63.7. I can understand that the latter scaffold is after curation. But readers may get confused. So kindly rephrase the sentence.

 2. A scale could have been included in the specimen photograph. If the author has any other phot graph with a scale for this specimen then the photograph can be replaced.

 3. Did the authors get mitogenome as a single scaffold? If yes, then that can be mentioned in this data article.

Are sufficient details of methods and materials provided to allow replication by others?

Yes

Is the rationale for creating the dataset(s) clearly described?

Yes

Are the datasets clearly presented in a useable and accessible format?

Yes

Are the protocols appropriate and is the work technically sound?

Yes

Reviewer Expertise:

Molecular Biology, Transcriptomics, Genomics, Regeneration biology

We confirm that we have read this submission and believe that we have an appropriate level of expertise to confirm that it is of an acceptable scientific standard, however we have significant reservations, as outlined above.

## References

[1] : A chromosome-level assembly of the harlequin ladybird Harmonia axyridis as a genomic resource to study beetle and invasion biology. *Mol Ecol Resour* .2021;21(4) : 10.1111/1755-0998.13342 1318-1332 10.1111/1755-0998.13342 33529495

# Reviewer report: Reviewer response for version 2

**DOI:** 10.21956/wellcomeopenres.23551.r105748

**Published:** 2024-11-01

**Authors:** Arun Sethuraman

**Affiliations:** 1 San Diego State University, San Diego, California, USA

I don't have any other comments.

Are sufficient details of methods and materials provided to allow replication by others?

No

Is the rationale for creating the dataset(s) clearly described?

Yes

Are the datasets clearly presented in a useable and accessible format?

Partly

Are the protocols appropriate and is the work technically sound?

Yes

Reviewer Expertise:

Genomics, population genetics, evolution

I confirm that I have read this submission and believe that I have an appropriate level of expertise to confirm that it is of an acceptable scientific standard.

# Reviewer report: Reviewer response for version 1

**DOI:** 10.21956/wellcomeopenres.19179.r56659

**Published:** 2023-05-31

**Authors:** Kang He

**Affiliations:** 1 Institute of Insect Science, Zhejiang University, Hangzhou, Zhejiang, China

In the present manuscript, Douglas *et al. *report a chromosome-level genome assembly of harlequin ladybird, I believe this dataset should be valuable for the comparative genomic study of species related. I only have several major concerns on this manuscript.

1. Given that a previous version of the chromosomal level genome assembly has been published (Chen *et al.*, 2021 ^1^), a lower BUSCO score of this version for the genome assembly is presented than the previous version. The authors are encouraged to explain the reason.
2. The 'Genome sequence report' mentions that the assembly sequence was assigned to 8 autosomes, including the X and Y sex chromosomes. However, in the method section, it is mentioned that the sequence data was from a single female H. axyridis. How can female's sequence data be assigned to the Y chromosome? Would males have DNA that the females don't have, and if so is this missing information in the assembly? Why not use male for sequencing?
3. The parameters in detail of the softwares for genome assembly are missing in the method part.

Are sufficient details of methods and materials provided to allow replication by others?

Partly

Is the rationale for creating the dataset(s) clearly described?

Yes

Are the datasets clearly presented in a useable and accessible format?

Yes

Are the protocols appropriate and is the work technically sound?

No

Reviewer Expertise:

Insect genomics, Non-conding RNA

I confirm that I have read this submission and believe that I have an appropriate level of expertise to state that I do not consider it to be of an acceptable scientific standard, for reasons outlined above.

## References

[1] : A chromosome-level assembly of the harlequin ladybird Harmonia axyridis as a genomic resource to study beetle and invasion biology. *Mol Ecol Resour* .2021;21(4) : 10.1111/1755-0998.13342 1318-1332 10.1111/1755-0998.13342 33529495

# Response

**Published:** 2024-10-10

**Authors:** Tree of Life Team Sanger

**Affiliations:** Wellcome Trust Sanger Institute, UK

Please see our responses to each part of the peer review report below. In the present manuscript, Douglas *et al. *report a chromosome-level genome assembly of harlequin ladybird, I believe this dataset should be valuable for the comparative genomic study of species related. I only have several major concerns on this manuscript.

1. Given that a previous version of the chromosomal level genome assembly has been published (Chen *et al.*, 2021), a lower BUSCO score of this version for the genome assembly is presented than the previous version. The authors are encouraged to explain the reason.

*Response*: The BUSCO score is actually **higher** when using the *insecta* dataset, as in Chen et al. (2021):

- Chen et al., 2021 reports 97.5% completeness, 1% fragmented (BUSCO v3, *insecta_odb9*).
- This data note (Boyes et al., 2021) reports 97.4% completeness, 0.6% fragmented (BUSCO v5.1.2, *endopterygota_odb10*). However, the BUSCO score for the assembly presented here is 99% when evaluated with *insecta_odb10* (see link: https://blobtoolkit.genomehubs.org/view/icHarAxyr1.1/dataset/CAJZBN01/busco).

 

1. The 'Genome sequence report' mentions that the assembly sequence was assigned to 8 autosomes, including the X and Y sex chromosomes. However, in the method section, it is mentioned that the sequence data was from a single female H. axyridis. How can female's sequence data be assigned to the Y chromosome? Would males have DNA that the females don't have, and if so is this missing information in the assembly? Why not use male for sequencing?

 
*Response*: This error has been corrected in the data note—no Y chromosome is present in this female specimen. A male was not used for sequencing because Darwin Tree of Life specimens are provided by collectors based on availability during collection events.

1. The parameters in detail of the softwares for genome assembly are missing in the method part.

*Response*: We have now included links to the protocols that detail how to run all the pipelines used in the Tree of Life genome assembly (see the Tree of Life bioinformatics pipelines at https://pipelines.tol.sanger.ac.uk/pipelines and https://github.com/sanger-tol/). **Is the rationale for creating the dataset(s) clearly described?** Yes **Are the protocols appropriate and is the work technically sound?** No *Response*: The genome assembly presented in this data note is the current reference genome for *Harmonia axyridis*, assembled using the standard Darwin Tree of Life pipeline. These methods are openly available, and all scripts can be downloaded (see links provided above). We also provide a link to the wet lab protocol collection at DOI:10.17504/protocols.io.8epv5xxy6g1b/v1. **Are sufficient details of methods and materials provided to allow replication by others?** Partly *Response*: We have now included links to comprehensive resources for replicating the methods used, including both wet lab protocols and bioinformatics pipelines. **Are the datasets clearly presented in a useable and accessible format?** Yes

# Reviewer report: Reviewer response for version 1

**DOI:** 10.21956/wellcomeopenres.19179.r48049

**Published:** 2022-02-03

**Authors:** Arun Sethuraman

**Affiliations:** 1 San Diego State University, San Diego, California, USA

The authors have presented a highly contiguous, high quality genome assembly for *Harmonia axyridis* from the UK. I am impressed at the completeness and contiguity of this assembly, and it just might be the most complete genome published for *H. axyridis* thus far. However, I think that this manuscript will benefit from: (1) comparison of assemblies with published ones out there, including Chen *et al*., 2021 ^1^; (2) sharing all software scripts/pipelines that were utilized in this assembly process (which are not currently available); and (3) additional details of assembly polishing utilized.

 Additionally, I acknowledge that this might be beyond the scope of this data note, but provision of a minimal annotation would be of great help to readers.

Are sufficient details of methods and materials provided to allow replication by others?

No

Is the rationale for creating the dataset(s) clearly described?

Yes

Are the datasets clearly presented in a useable and accessible format?

Partly

Are the protocols appropriate and is the work technically sound?

Yes

Reviewer Expertise:

Genomics, population genetics, evolution

I confirm that I have read this submission and believe that I have an appropriate level of expertise to confirm that it is of an acceptable scientific standard, however I have significant reservations, as outlined above.

## References

[1] : A chromosome-level assembly of the harlequin ladybird Harmonia axyridis as a genomic resource to study beetle and invasion biology. *Mol Ecol Resour* .2021;21(4) : 10.1111/1755-0998.13342 1318-1332 10.1111/1755-0998.13342 33529495
